# Radiographic appearance of proximal resin composite restorations bonded with a radiopaque or radiolucent universal adhesive: a randomized clinical trial

**DOI:** 10.1038/s41405-026-00494-1

**Published:** 2026-11-03

**Authors:** Omar Shaalan, Dina Kamal

**Affiliations:** Conservative Dentistry Department, Faculty of Dentistry, Cairo University, Cairo, Egypt

**Keywords:** Bonded restorations, Composite resin, Digital radiography in dentistry

## Abstract

**Objectives:**

This trial evaluated the radiographic appearance of resin composite restorations bonded with a radiopaque universal adhesive compared with a radiolucent universal adhesive, assessed by digital radiography based on modified USPHS criteria.

**Materials and methods:**

Seventy-four participants with class II cavities were randomly assigned to two groups (*n* = 37). The intervention group received radiopaque universal adhesive (Scotchbond Universal Plus, 3M ESPE, St. Paul, USA), while the control group received radiolucent universal adhesive (Single Bond Universal adhesive, 3M ESPE, St. Paul, USA). Selective enamel etching was performed, followed by adhesive application and restoration with nanofilled resin composite (Filtek Z350XT, 3M ESPE, St. Paul, USA). Postoperative periapical radiographs were used to evaluate the appearance of the tooth-restoration interface according to modified USPHS criteria, classifying restorations as Alpha (absence of radiolucent line) or Bravo (presence of radiolucent line). Categorical data were analyzed using Fisher’s exact test. Absolute risk reduction (risk difference) with 95% confidence interval was calculated to estimate the difference in the risk of detecting a radiolucent adhesive line between the two groups.

**Results:**

A statistically significant difference was observed between the two groups (*P* = 0.025). Restorations bonded with the radiopaque adhesive demonstrated a 16.2% absolute reduction in the risk of exhibiting a radiolucent line at the tooth-restoration interface compared with those bonded using the radiolucent adhesive (95% CI: 3.71–28.73%).

**Conclusions:**

The radiopaque universal adhesive resulted in significantly fewer restorations with visible radiolucent lines at the tooth-restoration interface immediately after restoration placement and may facilitate radiographic interpretation of proximal resin composite restorations.

**Trial Registration:**

https://clinicaltrials.gov/, (NCT05907928).

## Introduction

Overtreatment refers to the diagnosis and treatment of conditions that do not exist or the provision of care that exceeds a patient’s actual clinical needs. It is increasingly recognized as a significant concern in dentistry, where unnecessary interventions may result in the loss of healthy tooth structure and increased treatment costs [1]. One clinical scenario that may contribute to overtreatment is the radiographic detection of radiolucent areas beneath resin composite restorations. Distinguishing whether these radiolucencies represent secondary caries, marginal gaps, voids, or a radiolucent adhesive layer can be challenging. In many cases, the definitive diagnosis can only be established after removing the existing restoration. If the radiolucency is attributable solely to the adhesive layer, the restoration may be unnecessarily replaced despite the absence of underlying pathology [2].

Previous investigations have demonstrated that radiolucent adhesive layers may influence clinicians’ restorative decisions. An in vitro study reported that radiolucent adhesives were associated with a significantly higher frequency of incorrect radiographic decisions leading to restoration replacement [3]. Likewise, another study reported that dentists were approximately twice as likely to indicate replacement when a radiolucent area was observed beneath a composite restoration, even when the radiolucency resulted only from a thick adhesive layer [4].

Radiopacity is an important property of dental adhesive systems because it facilitates radiographic differentiation between the adhesive interface and radiolucent defects, such as marginal gaps, voids, and recurrent caries [5]. Traditionally, adhesive radiopacity has been achieved by incorporating inorganic radiopaque compounds or fillers, including barium-containing glass fillers, ytterbium trifluoride, and zirconium oxide, which increase X-ray attenuation and improve radiographic visibility [6]. Although these materials enhance radiopacity, they may influence adhesive viscosity, film thickness, filler sedimentation, and handling characteristics if not appropriately formulated [7, 8]. More recently, alternative strategies have been introduced in which radiopacity is incorporated directly into the resin matrix through radiopaque monomers rather than suspended inorganic fillers. Previous laboratory studies have shown that these formulations can achieve radiopacity comparable to dentin while maintaining favorable physicochemical and bonding properties [9].

A newly introduced universal adhesive (Scotchbond Universal Plus, 3M ESPE) was developed based on the chemistry of its predecessor (Single Bond Universal, 3M ESPE), with the incorporation of a radiopaque brominated dimethacrylate monomer into the adhesive resin matrix. This strategy was designed to improve radiographic visualization of the adhesive interface while maintaining the handling characteristics expected of universal adhesives [9]. Although the increased radiographic attenuation of a radiopaque adhesive would be expected from its material composition, in vitro radiopacity alone does not establish the magnitude or clinical detectability of this effect when the adhesive is applied within an actual tooth-restoration interface. Clinical factors such as adhesive application, film formation, localized pooling, cavity geometry, restorative material thickness, and radiographic projection may influence whether an interfacial radiolucency is detectable. Therefore, a clinical trial is required to determine whether the theoretical radiopacity advantage translates into a consistent and clinically observable difference in the radiographic appearance of proximal restorations.

However, evidence regarding the clinical radiographic performance of this newly developed adhesive remains limited. Therefore, the present randomized clinical trial aimed to evaluate the immediate radiographic appearance of the tooth-restoration interface of proximal resin composite restorations placed using either a radiopaque universal adhesive or a conventional radiolucent universal adhesive based on the modified USPHS radiographic criteria. The null hypothesis tested was that no difference would be observed between the two adhesive systems regarding the immediate radiographic appearance of the tooth-restoration interface.

## Participants and Methods

The materials used in this trial are described in Table 1 and were placed per the manufacturer’s instructions. All clinical procedures were carried out by a single operator with more than 15 years of experience in restorative dentistry. Before the trial commenced, the operator placed 10 restorations using each adhesive on patients who were not part of the study. To calibrate the two independent radiographic examiners, a separate set of radiographs not included in the trial was utilized. The examiners reviewed these training cases independently until a consensus agreement on the modified USPHS criteria scoring was achieved, ensuring uniform evaluation standards before study commencement.

**Table 1:** Commercial name, manufacturer, chemical composition, and lot number of the studied materials.

Commercial name	Manufacturer	Filler type	Filler content (weight)	Matrix composition	Lot number
Scotchbond Universal Plus	3M ESPE, St. Paul, USA	Synthetic amorphous silica, fumed, crystalline free, 2-propenoic acid, 2-methyl-, 3-(triethoxysilyl)propyl ester, reaction products with silica and 3-(triethoxysilyl)-1-propanamine (silica-based, silanated filler)	~5–15%	2-Propenoic acid, 2-methyl-, diesters with 4,6-dibromo-1,3-benzenediol, etc. (Brominated dimethacrylate), Hydroxyethyl methacrylate (HEMA), Diethylene glycol dimethacrylate (DEGDMA), Vitrebond copolymer, 10-methacryloyloxydecyl dihydrogen phosphate (MDP), Silane coupling agent, Camphorquinone, Ethanol, Water, Additives	6840217
Single Bond Universal	3M ESPE, St. Paul, USA	2-propenoic acid, 2-methyl-, 3-(trimethoxysilyl)propyl ester, reaction products with vitreous silica (silica-based, silanated filler)	~7–13%	Bisphenol A diglycidyl ether dimethacrylate (BISGMA), HEMA, (Dimethylamino)ethyl methacrylate, Vitrebond copolymer, MDP, Silane coupling agent, Camphorquinone, Ethanol, Water, Additives	527602
Scotchbond Etchant	3M ESPE, St. Paul, USA	N/A	N/A	Water, Phosphoric acid, Synthetic amorphous silica, Polyethylene glycol, Aluminum oxide (Concentration: 32%, pH: ~0.1%).	692513
Filtek Z350XT	3M ESPE, St. Paul, USA	Non‑agglomerated/non‑aggregated 20 nm silica filler, non‑agglomerated/non‑aggregated 4–11 nm zirconia filler, and aggregated zirconia/silica cluster filler (comprising 20 nm silica and 4–11 nm zirconia particles)	78.5%	BISGMA, Bisphenol A Polyethylene Glycol Diether Dimethacrylate (BISEMA-6), Diurethane Dimethacrylate (UDMA), Polyethylene Glycol Dimethacrylate (PEGDMA), Triethylene glycol dimethacrylate (TEGDMA)	NC93014

### Ethical approval and trial registration

The current trial was conducted and reported in accordance with the CONSORT 2010 guidelines. It was prospectively registered on clinicaltrials.gov (Registration number: NCT05907928, Date of first registration: 07/06/2023). All procedures involving human participants adhered to the ethical principles of the 2013 Declaration of Helsinki. Ethical approval was obtained from the Research Ethics Committee (REC) of Faculty of Dentistry, Cairo University, prior to participant recruitment (Approval ID: 57425).

### Sample size calculation

The sample size calculation was based on a previous study [10], which reported radiolucent interfacial lines beneath approximately 16% of proximal resin composite restorations. Assuming a two-sided significance level of 5%, a statistical power of 80%, and an expected between-group difference of 30%, the minimum required sample size was calculated to be 37 participants per group. The calculation was performed using G*Power software (version 3.1.9.2, Heinrich Heine University, Düsseldorf, Germany).

### Study design and setting

This study was a randomized, controlled clinical trial with a 1:1 allocation ratio, employing a parallel-group design under a superiority framework. Blinding was maintained for the participants, the independent radiographic assessors, and the statistician, all of whom remained unaware of the treatment group assignments. Blinding the clinical operator was not feasible due to perceptible differences in the physical handling and appearance of the adhesive materials.

The trial was conducted at the Conservative Dentistry clinics, Faculty of Dentistry, Cairo University, Egypt, from October 2025 to March 2026. Participant recruitment occurred between October and mid-December 2025, after which the treatment procedures continued until the end of March 2026. Recruitment commenced after obtaining the necessary institutional, ethical, and administrative approvals and procuring the specific dental materials and instruments required for the study. No unreported amendments to the original study protocol or predefined outcomes were made between trial registration and participant recruitment.

**Fig. 1:**
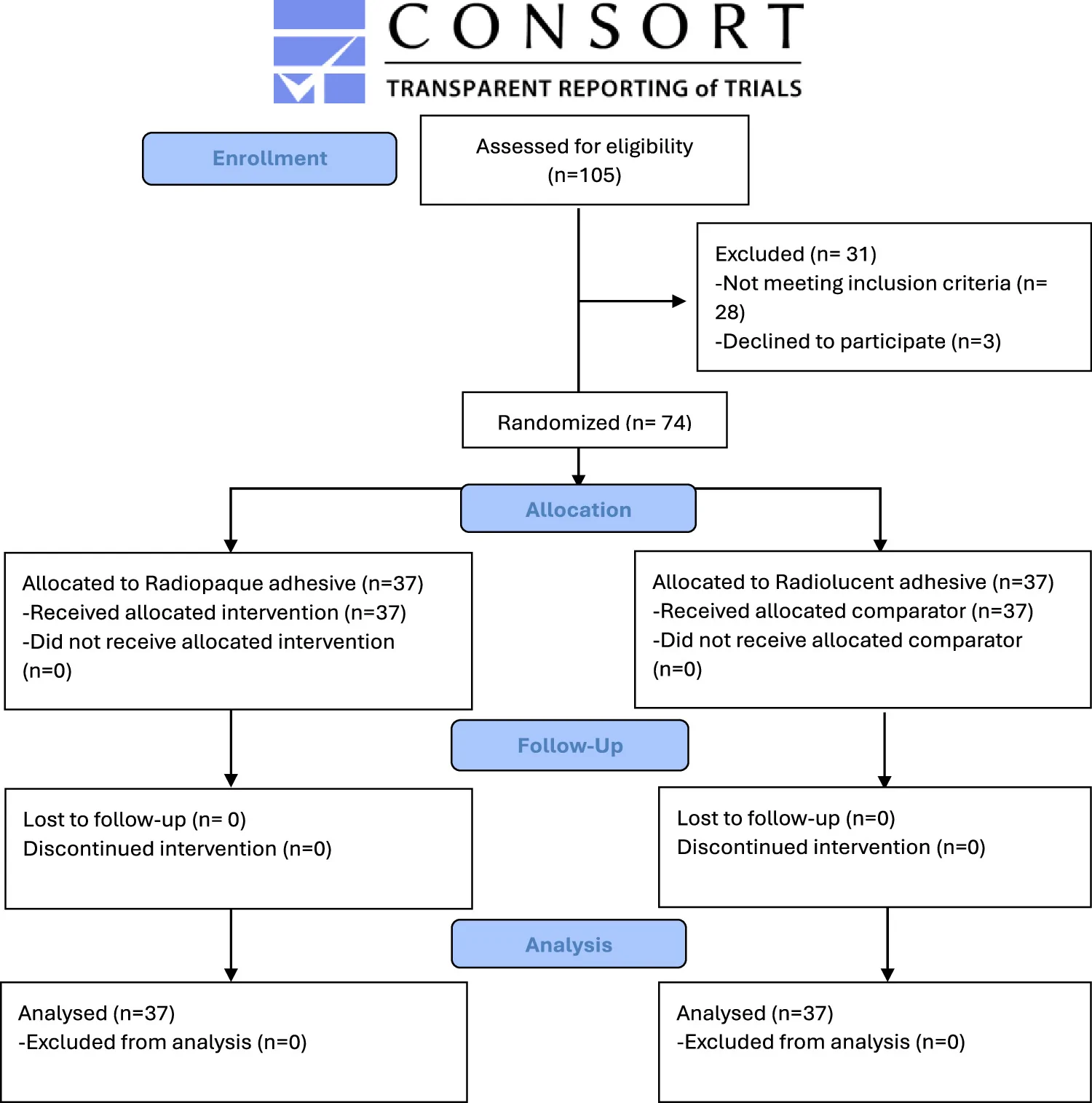
CONSORT Flow Diagram. Participant flow from enrollment through randomization, allocation, follow-up, and analysis (n = number of participants).

Participants were recruited using consecutive sampling. Eligible individuals were screened based on predefined inclusion and exclusion criteria. Before enrollment, all participants received comprehensive information about the procedures involved, including the number of visits, potential benefits, and any associated risks or harms. All participants provided written informed consent prior to their inclusion in the study. Seventy-four participants with posterior proximal carious cavities were randomly allocated to two groups (*n* = 37). The intervention group received resin composite restorations placed with a radiopaque universal adhesive, while the control group received restorations placed with a radiolucent universal adhesive. Radiographic interface appearance was assessed using digital radiography according to the modified USPHS criteria. The radiographic evaluation was performed immediately after restoration placement during the same clinical appointment. Participant flow is illustrated in the CONSORT flow diagram (Fig. 1).

### Eligibility criteria

Inclusion criteria included participants with small to moderate posterior proximal carious lesions (ICDAS scores 3 and 4); good oral hygiene; vital teeth with no signs of pulpitis; favorable occlusion and normal contact with adjacent teeth; and a healthy periodontium. Exclusion criteria included participants with systemic diseases or severe medical conditions; non-compliance; pregnancy; heavy smoking; xerostomia; teeth with deep carious lesions (within 1 mm of the pulp), signs or symptoms of irreversible pulpitis; pulp necrosis; endodontically treated teeth; dentin hypersensitivity; rampant caries; teeth indicated for prosthodontic restoration; advanced periodontal disease; evidence of parafunctional habits or TMJ disorders.

### Randomization, sequence generation, and allocation concealment

Simple randomization was performed using a computer-generated random allocation sequence (https://www.random.org/) to assign participants to either the intervention or control group. Allocation concealment was maintained by placing each assignment inside sequential, opaque, sealed envelopes. The allocation sequence was concealed from the investigators responsible for enrolling participants until the moment of assignment. The clinical operator opened each envelope only at the immediate time of the restorative procedure, ensuring that the treatment assignment could not be anticipated.

### Cavity preparation procedures

After local anesthesia administration, the tooth was isolated using a quadrant isolation technique with a rubber dam sheet (Sanctuary Dental Dam, Sanctuary Health Sdn Bhd, Perak, Malaysia) and appropriately sized clamps (KSK Clamps, Dentech Corporation, Tokyo, Japan). Class II cavity preparation was performed using sterile high speed #330 and #245 carbide burs (Hager & Meisinger GmbH, Neuss, Germany) mounted on a high-speed contra-angled handpiece (Alegra Handpiece, W&H, Bürmoos, Austria). Any soft carious tissue was removed using a sharp excavator of appropriate size (Zeffiro Excavator, Lascod S.p.A., Florence, Italy). Cavity width was standardized to no more than one-third of the intercuspal distance (moderate width), and the gingival seat was located at the equigingival or supragingival level [11]. Carbide burs were discarded and replaced after every five cavity preparations to ensure consistent cutting efficiency [12].

### Restorative procedures

A sectional matrix system (Composi-Tight 3D Fusion, Garrison Dental Solutions, Michigan, USA) was employed to restore the proximal wall. An appropriately sized sectional matrix band was positioned proximally, followed by insertion of a suitably sized diamond-shaped wedge into the lingual/palatal embrasure to ensure proper adaptation of the matrix band to the cavity margins. A separation ring was then applied using ring placement forceps. All materials were placed according to the manufacturers’ instructions. Selective enamel etching was performed using 32% phosphoric acid (Scotchbond Etchant, 3M ESPE, St. Paul, USA) for 15 seconds, followed by rinsing with a three-way syringe for 15 seconds and gentle drying with oil-free compressed air for 5 seconds. The adhesives, either Scotchbond Universal Plus or Single Bond Universal (3 MESPE, St. Paul, USA) were applied and actively rubbed onto the cavity surfaces using a microbrush for 20 seconds, followed by gentle air-thinning for 5 seconds. Light curing was performed for 10 seconds using an LED light-curing unit (iLED II, Guilin Woodpecker Medical Instrument Co., Ltd., Guangxi, China) with an intensity of 1200 mw/cm^2^ and a built-in radiometer, which was used to verify the light intensity every 10 uses. The prepared cavities were restored using a nanofilled resin composite (Filtek Z350XT, 3M ESPE, St. Paul, USA), employing the centripetal technique. The proximal wall was initially reconstructed by placing and light-curing a thin composite increment against the sectional matrix to convert the Class II cavity into a Class I configuration. The remaining cavity was then restored incrementally using oblique composite increments (2 mm/increment), each individually light-cured for 20 seconds. Occlusal evaluation was performed using double-sided articulating paper (#1012, Swedish Dental Supplies AB, Åkarp, Sweden) to identify premature contacts and cuspal interferences, which were adjusted sequentially. The restorations were finished using flame-shaped finishing diamond burs (Microdont, São Paulo, Brazil), followed by final polishing with a dedicated composite polishing kit (Resin polishers, Microdont, São Paulo, Brazil).

### Outcome assessment

Postoperative periapical radiographs were acquired using a size #2 digital sensor (KaVo Dental, Biberach, Germany) and an extension cone paralleling (XCP) film positioning device (TPC Advanced Technology, City of Industry, California, USA). Radiographic imaging was performed with an X‑ray unit (Sordex, Acteon Group, X‑mind DC, Rome, Italy) set at 70 kVp, 10 mA, and 0.08 seconds. Radiographic images were processed using Digora for Windows software through the Digora Optime system (Dexis, Pennsylvania, USA). The same radiographic protocol was applied to all participants to ensure standardized image acquisition.

Radiographic assessment was performed according to the modified United States Public Health Service (USPHS) criteria. An Alpha score indicated the absence of a visible radiolucent line at the tooth-restoration interface, whereas a Bravo score indicated the presence of a radiolucent line at the interface. Radiographs were independently evaluated by two blinded, trained assessors. In cases of disagreement, assessors discussed the findings to reach a consensus. No adverse events or unintended effects were observed during the study period.

### Statistical analysis

Data were analyzed using Medcalc software, version 22 for Windows (MedCalc Software Ltd, Ostend, Belgium). Categorical data were described as frequencies and percentages. Comparisons between categorical variables were performed using the Fisher’s exact test. All tests were two-tailed, and a P value ≤ 0.05 was considered statistically significant. Absolute risk reduction (risk difference) and its corresponding 95% confidence interval were calculated to estimate the difference in the risk of observing a radiolucent interfacial line between the two adhesive groups.

### Ethics approval and consent to participate

All procedures were conducted in accordance with the ethical principles of the 1975 Helsinki Declaration and its 2013 revision. Ethics approval was obtained from the Research Ethics Committee, Faculty of Dentistry, Cairo University (Approval ID: 57425). A written informed consent was obtained from all participants.

## Results

A total of 74 proximal resin composite restorations were placed in 74 participants, with one restoration evaluated per participant. The trial included 40 males (54%) and 34 females (46%). In the radiopaque adhesive group, 17 participants were male (45.9%) and 20 were female (54.1%), whereas in the radiolucent adhesive group, 23 were male (62.2%) and 14 were female (37.8%). No statistically significant difference was observed in gender distribution between the two groups (P = 0.1645). The distribution of restored teeth is presented in Table 2, with no statistically significant difference observed between the groups (P = 0.9411).

**Table 2:** Distribution of restored teeth among the two adhesive groups.

Teeth				
Adhesive	Maxillary premolars	Maxillary molars	Mandibular premolars	Mandibular molars
**Radiopaque Adhesive**	11	11	8	7
**Radiolucent Adhesive**	12	11	6	8
**Total**	23 (31.1%)	22 (29.7%)	14 (18.9%)	15 (20.3%)

**Fig. 2:**
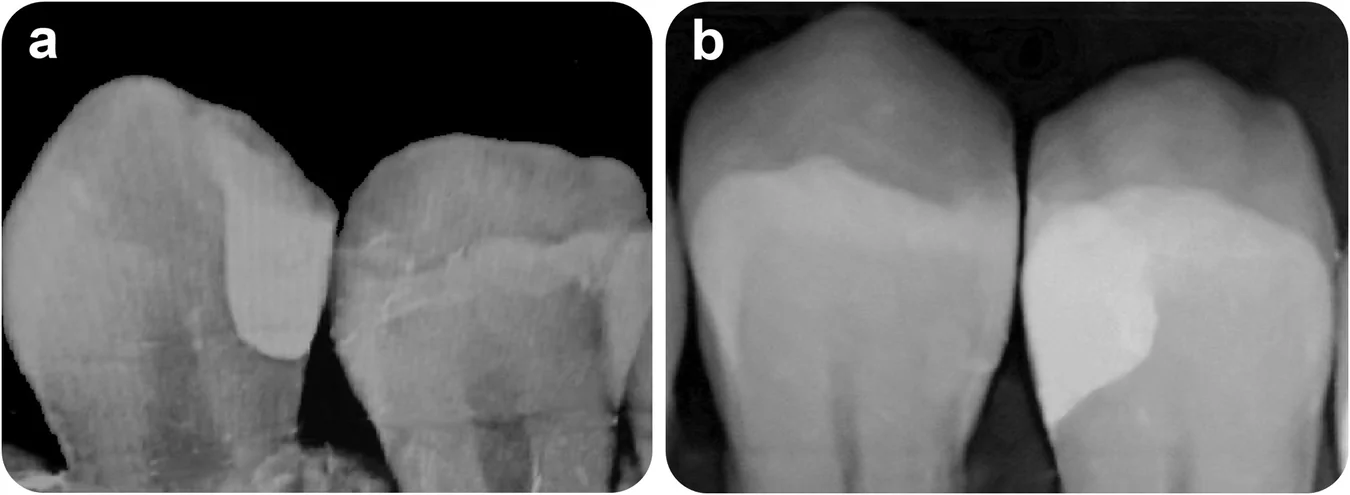
Representative postoperative radiographs of restorations placed using the two adhesive systems. **a** Restoration bonded with the radiolucent universal adhesive demonstrating a visible radiolucent line at the tooth-restoration interface (Bravo score). **b** Restoration bonded with the radiopaque universal adhesive demonstrating no visible radiolucent line at the tooth-restoration interface (Alpha score).

In the current study, 100% of restorations placed using the radiopaque adhesive received an Alpha (A) score, indicating the absence of a visible radiolucent line at the tooth-restoration interface. In contrast, 83.8% of restorations placed with the radiolucent adhesive received an Alpha (A) score, while 16.2% received a Bravo (B) score, indicating the presence of a visible radiolucent line at the tooth-restoration interface (Table 3, Fig. 2). The distribution of Alpha and Bravo scores differed significantly between the two adhesive groups (P = 0.025). The use of the radiopaque adhesive resulted in a 16.2% absolute reduction in the risk of observing a visible radiolucent line at the tooth-restoration interface compared with the radiolucent adhesive (95% CI: 3.71–28.73%). Inter-observer reliability for radiographic evaluation demonstrated almost perfect agreement between assessors (κ = 0.90).

**Table 3:** Distribution of modified USPHS radiographic scores according to adhesive group.

Adhesive	Radiographic evaluation	
	Alpha (A)	Bravo (B)
**Radiopaque Adhesive**	37 (100.0%)	0 (0.0%)
**Radiolucent Adhesive**	31(83.8%)	6 (16.2%)
**P value**	*P* = 0.025*	

*Statistically significant (*P* ≤ 0.05).

## Discussion

This randomized clinical trial aimed to evaluate the immediate radiographic appearance of proximal resin composite restorations bonded with either a radiopaque or a radiolucent universal adhesive. The results demonstrated a significantly higher proportion of Alpha scores in the radiopaque adhesive group compared with the radiolucent adhesive group, indicating the absence of a visible radiolucent interfacial line. Accordingly, the null hypothesis was rejected.

To the best of our knowledge, no previous randomized clinical trial has investigated the immediate radiographic appearance of proximal resin composite restorations bonded with radiopaque and radiolucent universal adhesives. The available literature has primarily focused on laboratory investigations evaluating the radiopacity and physicochemical properties of adhesive systems, as well as the influence of adhesive radiolucency on radiographic interpretation. The present randomized clinical trial therefore addresses this gap by providing clinical evidence on the radiographic appearance of restorations bonded with these two adhesive types.

The clinical relevance of adhesive radiopacity lies in its potential to reduce ambiguity when interpreting radiolucent areas beneath resin composite restorations. Radiolucent areas at the tooth-restoration interface may be associated with several conditions, including secondary caries, marginal gaps, voids, or the adhesive layer itself. When an adhesive layer is radiolucent, distinguishing between these possibilities may be difficult, particularly when radiographs are interpreted in isolation from the clinical examination [3, 13]. Previous investigations have demonstrated that radiolucent adhesive layers may increase the likelihood of recommending replacement of otherwise acceptable restorations [2, 4]. The present clinical findings extend these observations by demonstrating that the use of a radiopaque adhesive was associated with a lower frequency of visible radiolucent interfacial lines under clinical restorative conditions.

The observed difference may be explained, at least in part, by differences in the material composition and physical characteristics of the two adhesive systems. Adhesive viscosity, application behavior, localized pooling, and resulting film thickness may influence the radiographic visibility of an adhesive layer [3, 13]. Previous in vitro studies have shown that lower-viscosity adhesives may require the application of two consecutive coats to achieve optimal bond strength, whereas more viscous adhesive formulations may achieve comparable bonding performance with a single application. Clinically, multiple adhesive coats or localized adhesive pooling may increase adhesive film thickness, and a thicker layer of an inherently radiolucent adhesive may be more readily detected as an interfacial radiolucent line. Conversely, highly viscous formulations may exhibit reduced wettability, which could adversely influence adhesive infiltration and bonding performance [14, 15]. However, this represents a proposed explanatory mechanism rather than a directly tested hypothesis in the present study, as adhesive film thickness and interfacial morphology were not measured.

This interpretation is consistent with previous findings showing that Single Bond Universal exhibited a higher relative risk of radiolucent interfacial lines, which was attributed primarily to its viscosity [4]. Moreover, the adhesive layer thickness of Single Bond Universal was reported to be 15 ± 6 µm without adversely affecting bonding performance after six months of aging [16]. These findings provide contextual support for the possible influence of adhesive formulation and layer characteristics on radiographic appearance; however, they do not establish a causal relationship in the present study.

Beyond viscosity and application-related factors, the intrinsic radiopacity and formulation of the adhesive may also contribute to its radiographic appearance. Dental adhesives should exhibit sufficient radiopacity to allow radiographic differentiation from restorative defects and artifacts, ideally approaching the radiopacity of sound dentin [17]. Conventional inorganic radiopaque fillers may increase adhesive viscosity, potentially affecting wetting, handling, and infiltration. Furthermore, filler sedimentation may result in an inhomogeneous adhesive mixture unless the bottle is adequately shaken before use [3]. To avoid these potential limitations, radiopacity in the evaluated adhesive is chemically incorporated into the resin matrix rather than achieved primarily through conventional inorganic fillers. This approach is intended to provide radiopacity while maintaining the handling characteristics expected of universal adhesives and allowing homogeneous distribution of the radiopaque component throughout the adhesive [5, 17].

The observed Bravo scores indicate the presence of a visible radiolucent line at the tooth-restoration interface and should not automatically be interpreted as evidence of defective marginal adaptation, secondary caries, restoration failure, or inferior bonding performance. Therefore, the observed difference between the two adhesive systems should be interpreted as a difference in radiographic appearance rather than as evidence of a difference in bonding effectiveness, marginal integrity, or clinical performance. Longitudinal clinical assessment is required to determine whether the observed radiographic differences have any implications for restoration evaluation or clinical decision-making over time.

Digital radiography was used to evaluate the tooth-restoration interface, as systematic reviews and other studies have demonstrated acceptable diagnostic accuracy of radiographic methods for assessing the marginal integrity of proximal resin composite restorations [18–21]. Nevertheless, radiographic findings should always be interpreted in conjunction with the clinical examination.

Several limitations should therefore be considered. First, radiographic evaluation was performed immediately after restoration placement without longitudinal follow-up; therefore, the findings do not provide information regarding adhesive interface degradation, marginal breakdown, recurrent caries, or restoration longevity. Second, standardized periapical radiographs were used for outcome assessment. Although this imaging technique allowed standardized postoperative evaluation, bitewing radiographs are generally considered more appropriate for assessing proximal marginal integrity and may have greater sensitivity for detecting small interfacial defects. Third, the study relied exclusively on radiographic evaluation without a concurrent clinical assessment using the USPHS criteria. Accordingly, the findings should be interpreted as representing the immediate radiographic appearance of the tooth-restoration interface rather than overall restoration performance. Furthermore, the radiographic appearance of the tooth-restoration interface should not be interpreted as a direct measure of adhesive film thickness, marginal integrity, or bonding quality. Fourth, only one commercially available radiopaque universal adhesive and one commercially available radiolucent universal adhesive were evaluated. Therefore, the findings should not necessarily be generalized to all universal adhesive systems, as differences in formulation, radiopacity, viscosity, and handling characteristics may influence radiographic appearance. Future randomized clinical trials with longitudinal follow-up incorporating both clinical and radiographic outcome assessments are required to determine the long-term clinical relevance of radiopaque adhesive systems.

## Conclusions

The radiopaque universal adhesive produced fewer visible radiolucent lines at the tooth-restoration interface than the radiolucent universal adhesive when evaluated immediately after restoration placement. These findings suggest that radiopaque universal adhesives may facilitate radiographic interpretation of the tooth-restoration interface in proximal resin composite restorations by reducing the likelihood of visible interfacial radiolucent lines. However, these findings are limited to the immediate radiographic appearance and should not be interpreted as evidence of improved clinical performance or restoration longevity.

## Data Availability

The data supporting the findings of this study are available from the corresponding author upon reasonable request.
